# Differential Flo8p-dependent regulation of *FLO1* and *FLO11* for cell–cell and cell–substrate adherence of *S. cerevisiae* S288c

**DOI:** 10.1111/j.1365-2958.2007.06014.x

**Published:** 2007-12

**Authors:** Lars Fichtner, Florian Schulze, Gerhard H Braus

**Affiliations:** 1 Institut für Mikrobiologie und Genetik, Georg-August Universität Göttingen Grisebachstr8, D-37077 Göttingen, Germany.; 2 DFG Research Center for Molecular Physiology of the Brain (CMPB), Georg-August Universität Göttingen Grisebachstr8, D-37077 Göttingen, Germany.

## Abstract

Cell–cell and cell–surface adherence represents initial steps in forming multicellular aggregates or in establishing cell–surface interactions. The commonly used *Saccharomyces cerevisiae* laboratory strain S288c carries a *flo8* mutation, and is only able to express the flocculin-encoding genes *FLO1* and *FLO11*, when *FLO8* is restored. We show here that the two flocculin genes exhibit differences in regulation to execute distinct functions under various environmental conditions. In contrast to the laboratory strain Σ1278b, haploids of the S288c genetic background require *FLO1* for cell–cell and cell–substrate adhesion, whereas *FLO11* is required for pseudohyphae formation of diploids. In contrast to *FLO11*, *FLO1* repression requires the Sin4p mediator tail component, but is independent of the repressor Sfl1p. *FLO1* regulation also differs from *FLO11*, because it requires neither the *KSS1* MAP kinase cascade nor the pathways which lead to the transcription factors Gcn4p or Msn1p. The protein kinase A pathway and the transcription factors Flo8p and Mss11p are the major regulators for *FLO1* expression. Therefore, *S. cerevisiae* is prepared to simultaneously express two genes of its otherwise silenced *FLO* reservoir resulting in an appropriate cellular surface for different environments.

## Introduction

Natural fungal populations respond to appropriate environmental conditions by cell to cell adherence, cell to substrate adherence, or the formation of biofilms. Morphological changes such as flocculation or biofilm formation are important for various biotechnological processes. Adherence to human tissue and to plastic devices are of medical relevance because they represent initial steps in the establishment of pathogenic fungal–host interactions which can result in access to internal organs for the fungus. The budding yeast *Saccharomyces cerevisiae* has been used as a fungal model organism to explore cell–substrate and cell–cell adhesion. Diploid yeast strains are dimorphic and can therefore switch between a single celled and a filamentous pseudohyphal growth form with elongated cells. Diploid pseudohyphae formation depends on sufficient supply of fermentable carbon sources like glucose and simultaneous limitation of nitrogen sources such as ammonium ions (Gimeno and Fink, 1992; Gimeno *et al*., 1992; Mösch and Fink, 1997). In haploid yeast cells, adhesive growth, adherence to surfaces (Roberts and Fink, 1994; Guo *et al*., 2000) and formation of biofilms (Roy *et al*., 1991; Cappellaro *et al*., 1994; Reynolds and Fink, 2001) can be induced on rich media when carbon sources become limiting.

All adherence events require the expression of specific cell surface glycoproteins, which are encoded by the *FLO* gene family. In *S. cerevisiae*Σ1278*b*, which has been primarily used for such studies, only *FLO11* is expressed and is activated under specific environmental conditions (Guo *et al*., 2000; Halme *et al*., 2004; Verstrepen *et al*., 2004). Four additional *FLO* genes (*FLO1*, *FLO5*, *FLO9* and *FLO10*) are epigenetically silenced by different histone deacetylases (HDAC) (Halme *et al*., 2004). The active *FLO11* gene, which is also named *MUC1* (Lambrechts *et al*., 1996), encodes a glycosyl-phosphatidylinositol-linked glycoprotein similar to adhesins of pathogenic fungi (Lo and Dranginis, 1996).

The inducible *FLO11* gene carries one of the largest promoters of the yeast genome (Rupp *et al*., 1999). Various transcription factors, which perceive multiple distinct external signals from specific signal transduction cascades (Banuett, 1998; Lengeler *et al*., 2000). During the yeast form of growth, *FLO11* expression is inhibited by HDAC silencing and by repressors such as Sfl1p (Pan and Heitman, 2002) or Nrg1p/Nrg2p (Kuchin *et al*., 2002) which interact with the *FLO11* promoter. The corepressor Tup1p which has multiple functions in yeast acts in concert with Sfl1p and also affects the Nrg repressors (Berkey *et al*., 2004). Various components of the RNA polymerase II mediator complex including Sin4p (Conlan and Tzamarias, 2001), Srb8p or Ssn8p, have been identified as additional repressors for *FLO11* expression (Palecek *et al*., 2000).

Glucose starvation in haploids or nitrogen starvation in diploids overcomes *FLO11* repression, and aromatic alcohols have been identified as inducing signals (Chen and Fink, 2006).

Several signalling pathways, including the mitogen-activated protein kinase (MAPK) cascade with Kss1p as specific MAP kinase or the cAMP-dependent protein kinase A (PKA) pathway activate *FLO11* expression (Roberts and Fink, 1994; Mösch *et al*., 1999; Rupp *et al*., 1999; Cullen and Sprague, 2000; Palecek *et al*., 2000). Kss1p activates the transcription factors Ste12p and Tec1p, and PKA activates the transcription factor Flo8p (Köhler *et al*., 2002; Brückner *et al*., 2004; Chou *et al*., 2004). Mss11p represents another transcriptional activator that plays an essential role at the convergence of the MAPK and PKA pathways (Gagiano *et al*., 1999; 2003; van Dyk *et al*., 2005). The repressor Sfl1p and the activator Flo8p antagonistically control the expression of *FLO11* by binding to a common promoter element. Sfl1p and Flo8p are direct molecular targets of the PKA catalytic subunit Tpk2p. Phosphorylation by PKA promotes Flo8p binding and activation of the *FLO11* promoter and relieves repression by prohibiting dimerization and DNA binding by Sfl1p (Pan and Heitman, 2002). Low glucose in haploid and low nitrogen in diploid yeasts also activate the protein kinase Snf1p which positively regulates *FLO11* expression by antagonizing the two repressors Nrg1p and Nrg2p (Kuchin *et al*., 2002).

*FLO11* expression can also respond to other forms of nutritional limitations including amino acid starvation in haploid as well as in diploid cells. This response depends on the transcription factor Gcn4p which is regulated by the general control of amino acid biosynthesis pathway and its sensor kinase Gcn2p (Hinnebusch, 1997; Hinnebusch and Natarajan, 2002; Braus *et al*., 2003). The large *FLO11* promoter is also affected by additional factors which support adaptation to changing environmental conditions including the transcription factors Sok2p (Pan and Heitman, 2000; Vachova *et al*., 2004), Phd1p (Gimeno and Fink, 1992) and the product of the *MSN1* (also known as *PHD2*, *MSS10* or *FUP4*) gene (Lorenz and Heitman, 1998). In addition to the transcriptional regulation, there is also evidence for control of *FLO11* expression on a post-transcriptional level (Strittmatter *et al*., 2006).

In industrial yeasts, including flocculent bottom-fermenting yeast strains, another gene of the *FLO* family, *FLO1*, has been shown to be active and regulated by *FLO8*. It is considered to play an important role in mannose-specific flocculation, which is inhibited by mannose but not by glucose (Kobayashi *et al*., 1996; 1998; 1999). In *S. cerevisiae*Σ1278*b*, *FLO1* is silenced and has only artificially been activated by inserting the *GAL1* promoter upstream of the *FLO1* open reading frame. Induced expression of this engineered *GAL1-FLO1* strain in galactose medium also resulted in enhanced flocculation (Guo *et al*., 2000; Halme *et al*., 2004; Verstrepen *et al*., 2004). The most commonly used *S. cerevisiae* laboratory strain S288c is impaired in haploid adhesion, biofilm formation and diploid pseudohyphal growth. This has been at least partially attributed to the acquisition of a nonsense mutation in the *FLO8* gene encoding one of the key transcriptional activators of *FLO* genes (Liu *et al*., 1996). Restoration of *FLO8* resulted in the activation of transcription of two *FLO* genes, *FLO11* and *FLO1*, suggesting a similar regulatory mechanism for both promoters (Kobayashi *et al*., 1999). In addition, overexpression of the transcription factor encoding *MSS11* (Bester *et al*., 2006) or the *GTS1* gene for a Sfl1p repressor interacting protein (Shen *et al*., 2006) induce *FLO1* transcription in *flo8*-deficient yeasts.

We have studied the regulation and the function of *FLO11* and *FLO1* in more detail in a *FLO8*-restored *S. cerevisiae* strain S288c. We find that Flo11p is primarily required to establish a cell–substrate interaction of the initial cell layer, whereas Flo1p is essential to support cell–cell interactions of the following cell layers of the colony. In addition, we find that the *FLO1* promoter is significantly less complex than the *FLO11* promoter. Laboratory conditions favour yeast strains which are less adhesive, because a detailed analysis of the *FLO1* locus of the Σ1278*b* strain revealed that the Flo8p binding site is missing in the promoter which is presumably the reason why only the *FLO11* gene can be activated in this yeast.

## Results

### *FLO1* and *FLO11* of S288c play distinct roles in adhesion, pseudohyphae formation and flocculation

**Fig. 1:**
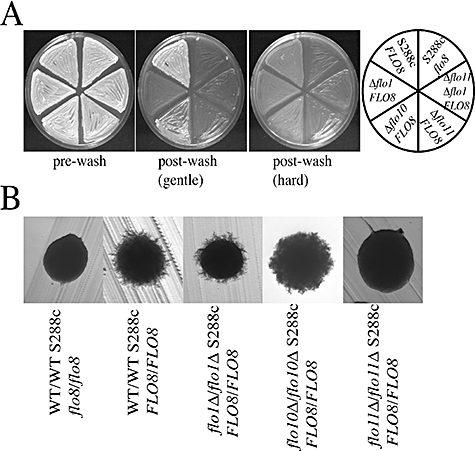
*FLO11* requirement for haploid and diploid *S. cerevisiae* S288c [*FLO8*]. A. Adhesion of *S. cerevisiae* S288c derivatives without (*flo8*) or with an intact integrated *FLO8* gene (pHL11) in response to glucose starvation. Indicated yeast strains of the S288c background were patched on YPD plates and incubated for 3 days at 30°C and subsequently for three more days at room temperature resulting in glucose starvation (pre-wash). Non-adhesive cells were first gently washed off the agar by agitating them in a bowl of water and then documented; subsequently they were placed under a hard stream of water (hard washing) and documented. Strains able to invade the agar show a remaining cell-layer in the agar surface even after the final washing step. B. Pseudohyphal growth of diploid homozygous *S. cerevisiae* S288c without (*flo8*) or with integrated *FLO8* genes in comparison with deletion derivatives carrying the indicated *flo* deletions. Homozygous diploid yeast strains as indicated were streaked on SLAD media and incubated at 30°C. Pseudohyphal growth of colonies was monitored after 6 days.

Haploid invasive growth of *S. cerevisiae*Σ1278*b* due to the induction of *FLO11* can be achieved in rich yeast peptone dextrose (YPD) medium after glucose becomes limiting which requires six or more days (Roberts and Fink, 1994; Guo *et al*., 2000). A comparison of different S288c [*FLO8*] derivatives revealed subtle differences in the intensity of adhesion, which are achieved by a cooperation of the two adhesins Flo1p and Flo11p (Fig. 1A). S288c [*FLO8*] cells carrying only a *flo11* deletion were completely washed off the agar surface as fluffs after a short time of gentle washing. Most of the corresponding *flo1*Δ derivative cells were also easily washed off the plate; however, a layer of yeast cells remained directly on the agar surface and could be washed off neither by a strong stream of water (Fig. 1A) nor by rubbing the surface (not shown). Strains lacking both flocculin genes, *FLO1* and *FLO11*, are completely washed off the agar without any remaining cell layer on the surface. This suggests that under these glucose limiting conditions both flocculins are required and fulfil distinct functions for S288c [*FLO8*]. Flo11p seems to be required for cell–substrate interaction for anchoring the yeast population to the agar surface. Flo1p allows cell–cell interaction of different yeast cell layers, which resembles its function during flocculation. The Flo11p substrate interaction function is not necessary for adhesion to synthetic complete (SC) medium, to plastic surfaces and for flocculation where Flo1p alone is crucial and sufficient. In Σ1278*b*, such a functional diversity of flocculins has not been described, and all known aspects of adhesive growth are contributed exclusively to Flo11p.

*FLO11* is also required for pseudohyphae formation of diploid yeast Σ1278*b*, which can be induced when sufficient carbon sources are available but ammonium ions are limiting. Pseudohyphae formation of S288c requires an intact *FLO8* gene (Fig. 1B). When we analysed the formation of pseudohyphae of diploid S288c [*FLO8*] derivatives carrying homozygous *flo11* deletions, we found that they showed the same morphological phenotypes as described for Σ1278*b* mutant strains (Gimeno and Fink, 1992; Mösch and Fink, 1997). Like in the Σ1278*b* background, diploid S288c [*FLO8*] strains deleted for both copies of *FLO11* are not able to form pseudohyphae under inducing conditions. However, S288c [*FLO8*] derivatives carrying homozygous *flo1* or *flo10* deletions are not impaired in pseudohyphae formation.

**Fig. 2:**
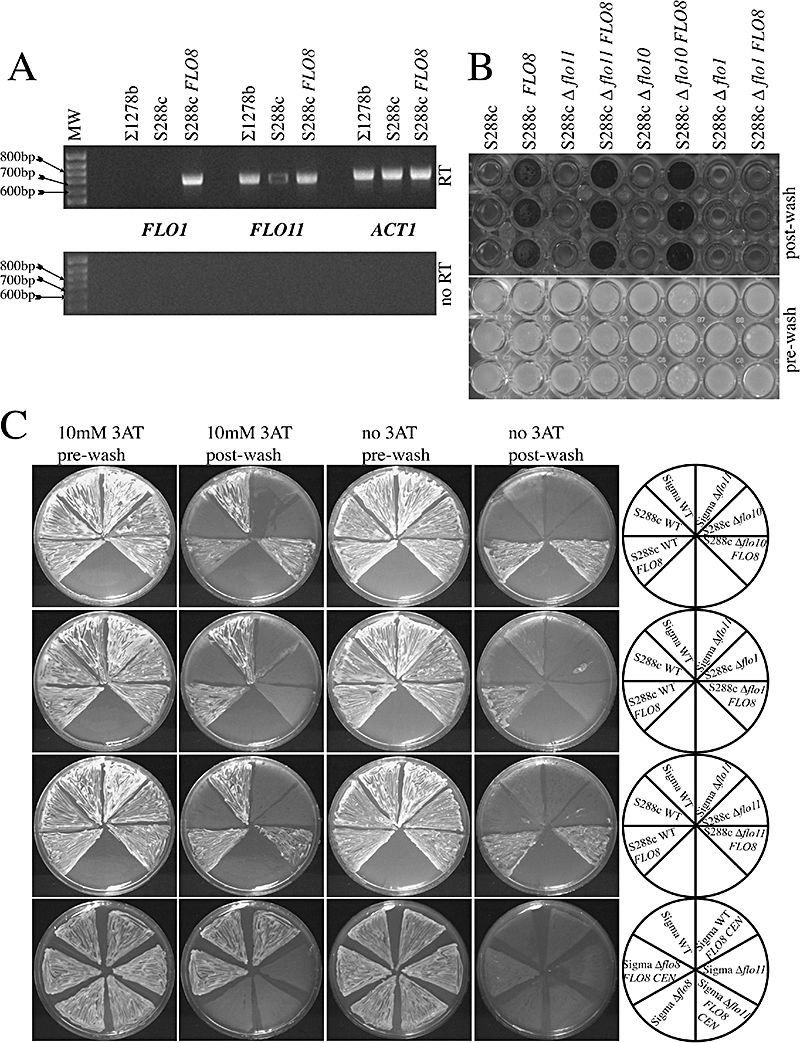
Induction of *FLO* genes of haploid *S. cerevisiae* S288c [*FLO8*] in comparison with haploid *S. cerevisiae*Σ1278*b*. A. *FLO1* and *FLO11* transcript levels of yeast S288c, S288c [*FLO8*] (pHL11) and Σ1278*b* respectively. RNAs of the various strains grown on SC media were isolated and compared after RT-PCR followed by semiquantitative PCR using *Taq* polymerase. The PCR reactions were compared by 1% TAE-agarose gel electrophoresis. *ACT1* transcript levels served as control. B. Adhesion to plastic surfaces of *S. cerevisiae* S288c derivatives. Ninety-six well plate with indicated yeast strains grown for 24 h in liquid SC media are documented before and after staining and washing. After staining cells with crystal violet and subsequent washing, biofilm formation on the plastic surface is indicated by a remaining cell-layer (dark wells). C. Yeast S288c, S288c [*FLO8*], Σ1278*b* (adhesive control) and Σ1278*b flo11*Δ (non-adhesive control) are compared with S288c *flo10*Δ with or without intact *FLO8* gene (first row), with S288c *flo1*Δ with or without intact *FLO8* gene (second panel) and with S288c *flo11*Δ with or without intact *FLO8* gene (third row). The fourth panel compares adhesive growth of indicated Σ1278*b* strains with or without an extra copy of the *FLO8* gene (pHL1). Adhesion was assayed after 1 day of growth on SC medium without histidine and on amino acid starvation-inducing SC medium caused by the addition of the histidine analogue 3-amino-triazole (3AT). Plates were documented before and after non-adhesive cells were washed off the agar.

We then analysed adhesion on plastic surfaces in SC medium of several haploid S288c [*FLO8*] derivatives to further explore the role of *FLO1* for haploid adhesive growth of this strain in more detail (Fig. 2B). Neither the deletion of *FLO11* nor the deletion of another *FLO* gene, *FLO10*, abolished the capability for adhesion of a S288c strain with an intact *FLO8* gene. However, the deletion of *FLO1* resulted in non-adherent yeasts in spite of an intact *FLO8* which is comparable to all S288c derivatives without intact *FLO8* genes. In contrast to Σ1278*b*, this further corroborates that under these conditions *FLO1* and not *FLO11* is the major adhesin for substrate interaction of S288c [*FLO8*].

These pronounced differences in the induction of haploid adhesive growth between S288c [*FLO8*] and Σ1278*b* prompted us to a further comparative analysis. Induction of adhesion of Σ1278*b* can be achieved in SC medium by amino acid starvation; therefore, the addition of the histidine analogue 3-amino-triazole (3AT) results in the expression of *FLO11* in Σ1278*b* (Braus *et al*., 2003). Σ1278*b* only shows the adherence phenotype in SC medium in the presence of 3AT whereas cultivation of S288c [*FLO8*] on SC media with or without supplementation of 10 mM 3AT resulted in adhesion (Fig. 2C). We transformed a Σ1278*b* strain with additional copies of *FLO8* (pHL1) to exclude any dosage effect of the transcriptional activator gene. However, the transformed Σ1278*b* strain is still unable to grow adhesively in the absence of 3AT. These data further suggest that *FLO1*-dependent adhesion of S288c [*FLO8*] is differently regulated in comparison with *FLO11*-regulated adhesion of Σ1278*b*.

We analysed several S288c [*FLO8*] derivatives to further address differences in *FLO* regulation. S288c [*FLO8*] strains deleted for *FLO1* (*flo1*Δ) failed to grow adhesively under every condition tested. Correspondingly, *flo10* or *flo11* deletion strains exhibited a similar phenotype as S288c [*FLO8*] and therefore do not seem to play a major role during haploid adhesion under these conditions. These data further corroborate that in contrast to the yeast Σ1278*b*, Flo1p plays a prominent and specific role for haploid adherence of S288c [*FLO8*] yeast cells.

The importance of *FLO1* was further analysed by flocculation assays in liquid culture of haploid S288c [*FLO8*] derivatives carrying deletions in *flo1*Δ, *flo10*Δ and *flo11*Δ respectively, in comparison with Σ1278*b*(Table 1). Flocculation reflects the potential for cell–cell interactions. In the Σ1278*b* background, a haploid *flo11*Δ strain does not flocculate. For Flo8p-dependent flocculation of S288c [*FLO8*], *FLO1* is exclusively essential, whereas *flo10*Δ and *flo11*Δ strains flocculated similarly as the S288c [*FLO8*] control. This suggests an additional crucial role of Flo1p for cell–cell interactions in haploid yeast S288c [*FLO8*].

**Table 1:** Flocculation of *flo*Δ strains.

	Σ1278*b*	Σ1278*b flo11*Δ	S288c	S288c *flo11*Δ	S288c *flo1*Δ	S288c *flo10*Δ
Without *FLO8*	–	–	–	–	–	–
	FA = 0.002	FA = 0.007	FA = 0.004	FA = 0.011	FA = 0.006	FA = 0.009
With *FLO8*	+	–	+	+	–	+
	FA = 0.102	FA = 0.013	FA = 0.532	FA = 0.399	FA = 0.02	FA = 0.584

Flocculation was assayed in SC media. The presence or absence of flocculation was visually checked (+/–) and quantified (Kobayashi *et al*., 1996). FA values represent the average of three independent measurements. *FLO8* was reconstituted in S288c strains by integration of pHL11. Σ1278b without *FLO8* corresponds to *flo8*Δ.

In summary, these results demonstrate that the haploid yeast S288c [*FLO8*] requires *FLO1* for adhesive growth on substrates and cell–cell interactions during flocculation, two functions which are fulfilled by *FLO11* in Σ1278*b*. In addition, the amino acid starvation experiments suggest differences in the regulation of *FLO1* and *FLO11.* For diploid pseudohyphae formation in S288c, like in Σ1278*b*, Flo11p is the only essential cell–surface protein.

### *FLO1* and *FLO11* are differentially regulated in the commonly used *S. cerevisiae* S288c *[FLO8]* strain

**Fig. 3:**
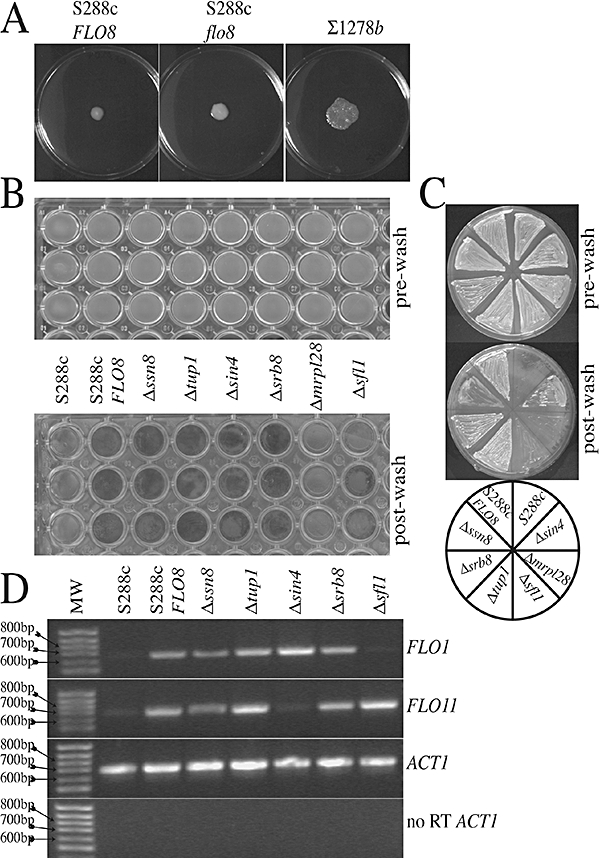
Genetic suppression analysis of the *S. cerevisiae* strain S288c *flo8* mutant allele. A. Biofilm formation. *S. cerevisiae* S288c (*flo8*), S288c with intact *FLO8* copies (on the high copy number plasmid pHL135) and Σ1278*b* were grown for 4 days on 0.3% agar, 0.2% glucose YPD media. The formation of mats was documented. B. Adhesion to plastic surfaces of *S. cerevisiae* S288c derivatives. S288c (*flo8*) and S288c carrying an integrated intact copy of *FLO8* (S288c [*FLO8*]) are compared with S288c derivatives carrying the indicated suppressor mutations in addition to the *flo8* mutant allele. Suppressor strains were identified by screening the entire yeast collection of nonessential gene deletions. Strains were grown on 96 well plates for 24 h in liquid SC media (upper panel), stained by crystal violet and washed (lower panel). Adhesive growth is visible as a remaining cell-layer after washing. C. Adhesion to agar surfaces of *S. cerevisiae* S288c derivatives. The same strains as in (B) were patched on YPD plates and incubated for 3 days at 30°C and subsequently for three more days at room temperature. The plate was documented before non-adhesive cells were washed off the agar as well as after gentle washing by agitating it in a bowl of water. D. Expression of the adhesin-encoding *FLO1* and *FLO11* genes of *S. cerevisiae* S288c derivatives. *FLO1* and *FLO11* mRNA levels of the indicated strains were compared by RT-PCR followed by semiquantitative PCR using *Taq* polymerase. *ACT1* transcripts were used as standard and MW indicates the sizes.

As shown before, *FLO1* and *FLO11* of the commonly used *S. cerevisiae* S288c can only be activated when the defective allele for the transcription activator Flo8p is restored (Liu *et al*., 1996; Kobayashi *et al*., 1999). Haploid adhesion to substrates and diploid filamentous growth can be regained by *FLO8*, whereas biofilm formation cannot be restored even in high copy number (Fig. 3A). It had been proposed that *FLO1* might be similarly regulated as *FLO11* (Kobayashi *et al*., 1999). To compare the regulation of both genes in more detail, we performed a genetic screen for suppressor mutations of *flo8*, resulting in haploid adhesive growth of S288c.

Individual knockout strains of the ordered S288c yeast deletion collection (Brachmann *et al*., 1998) were grown in liquid SC medium in microtiter plates and assayed for their ability to adhere to the plastic surface. Six mutant strains were able to suppress the adherence defect of yeast S288c on plastic surfaces (Fig. 3B) and were verified by assaying adherence on SC agar for cells growing on Petri dishes (Fig. 3C). The *mrpl28*Δ mutant strain was not included into further analyses, because it showed a significantly weaker adhesion phenotype than the others. Whereas the *sfl1*Δ mutation resulted only in partial suppression, the *tup1*Δ, *ssn8*Δ, *sin4*Δ and *srb8*Δ deletions were strong suppressor mutations of adhesive growth (Fig. 3B and C). Sfl1p is bridged to Tup1p and requires the mediator tail Sin4p to be one of the major specific repressors of the adhesin gene *FLO11* of *S. cerevisiae*Σ1278*b* (Conlan and Tzamarias, 2001). Srb8p and Ssn8p are additional mediator components which repress *FLO11* expression (Palecek *et al*., 2000). We were surprised that in S288c, the impact of *tup1*Δ or the three mediator genes on adhesive growth was significantly more pronounced than the impact of a *sfl1*Δ deletion (Fig. 3B and C). This suggests that Tup1p and the mediator components might have additional Sfl1p-independent functions in repressing adherence of S288c.

For a more detailed analysis, we wanted to know which *FLO* gene is affected by the five suppressor mutations that restore adhesive growth of *S. cerevisiae* S288c. Figure 3D shows that integration of an intact *FLO8* activator gene induces *FLO11* and *FLO1* in S288c as expected, whereas strain Σ1278*b* is only able to activate *FLO11* (Fig. 2A). None of the suppressors resulted in the induction of the silent *FLO5*, *FLO9* or *FLO10* (data not shown). Deletion of *SFL1* in the *flo8* genetic background, which only partially restores adhesive growth, resulted only in the induction of *FLO11*. However, defects in the gene for the mobile repressor Tup1p resulted in the induction of both *FLO11* and *FLO1*. This suggests that Tup1p is involved not only in Sfl1p-mediated *FLO11* repression but also in Sfl1p-independent *FLO1* repression. Defects in the mediator genes for Srb8p and Ssn8p also resulted in the induction of both *FLO* genes, suggesting a similar mode of repression for both genes. Interestingly, the deletion of the strong suppressor gene *sin4*Δ encoding a part of the mediator tail resulted only in the induction of *FLO1* but not of *FLO11* (Fig. 3D). The result that the *sin4* mutation represents a significantly stronger suppressor gene of the adherence defect of S288c than the *sfl1* deletion points to a more prominent role of *FLO1* than *FLO11* under these conditions for S288c adhesion.

In summary, these data further support that *FLO1* is even more important than *FLO11* for haploid adherence under specific growth conditions in the yeast strain S288c, which carries a restored *FLO8* gene. *FLO1* and *FLO11* regulation share similarities but there are also significant differences in regulation, because *FLO1* depends on not only an active mediator but also the mediator tail protein Sin4p. In addition, induction of *FLO1* in yeast S288c is independent of Sfl1p but not Tup1p.

**Fig. 4:**
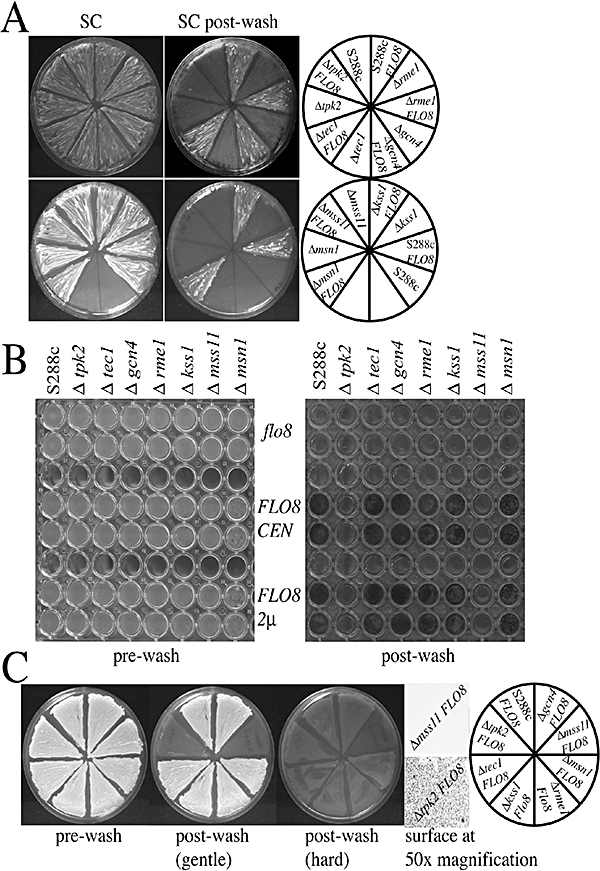
Haploid adhesive growth of *S. cerevisiae* S288c [*FLO8*] derivatives is impaired in various gene deletions of the filamentous growth pathway. A. Haploid adhesive growth on SC agar plates. Indicated yeast strains were grown for 1 day on SC media. The plates were documented before and after non-adhesive cells were washed off the agar. B. Haploid adhesive yeast growth in liquid cultures. Ninety-six well plates with indicated yeast strains were grown for 24 h in liquid SC media (pre-wash) and the same 96-well plate were washed after cell-staining with crystal violet (post-wash). Adhesive growth is indicated by a remaining cell-layer (dark wells) after washing. C. Haploid adhesive yeast growth after glucose starvation. Indicated S288c [*FLO8*] (pHL11) derivatives were patched on YPD plates and incubated for 3 days at 30°C and then stored for three more days at room temperature to induce glucose starvation. The plates were documented before non-adhesive cells were washed off the agar as described in Fig. 3. Strains able to invade the agar show a remaining cell-layer on the agar surface even after the final hard washing step. An example of a 50× magnification of cells on the agar surface after the last washing step is shown for yeast S288c [*FLO8*]*tpk2*Δ as an example in contrast to the *mss11*Δ cells which are completely washed away.

In Σ1278*b* yeast strains, Flo8p is activated by the cAMP-dependent PKA pathway and is impaired by a deletion of the *tpk2*Δ gene encoding the catalytic subunit of the PKA complex (Roberts and Fink, 1994; Mösch *et al*., 1999; Rupp *et al*., 1999; Cullen and Sprague, 2000). Here we show that a *tpk2*Δ deletion also prevents Flo8p-mediated haploid adhesive growth of S288c strains on agar (Fig. 4A), plastic (Fig. 4B) and rich medium after glucose starvation (Fig. 4C) respectively. Even *FLO8* overexpression from a high copy number plasmid could not suppress the *tpk2*Δ-mediated adherence defect and therefore the dependence for activation of the pathway by Tpk2p (Fig. 4B). Therefore, *FLO1* expression of S288c shares a similar control to *FLO11* by the PKA signal transduction pathway which activates the transcription factor Flo8p.

In parallel to the PKA pathway, the MAPK cascade has been shown to be crucial for *FLO11*-mediated haploid and diploid filamentous growth of Σ1278*b* (Roberts and Fink, 1994; Mösch *et al*., 1999; Rupp *et al*., 1999; Cullen and Sprague, 2000). However, neither the deletion of the gene for MAPK, *kss1*Δ, nor the deletion of *tec1*Δ encoding the corresponding *FLO11-*activating transcription factor impaired haploid invasive growth of S288c [*FLO8*] (Fig. 4). This suggests a less prominent role for the MAPK pathway in controlling *FLO1* and *FLO11* in S288c in comparison with Σ1278*b*.

Deletion analysis also showed that Flo8p-mediated adhesive growth of S288c requires neither the gene for the repressor of meiosis encoded by *RME1* (Gagiano *et al*., 2003) nor the transcription factor encoded by *MSN1* (Lorenz and Heitman, 1998) which have been both described to regulate *FLO11* expression of Σ1278*b*. The deletions Δ*rme1* or *msn1*Δ did not impair haploid adhesive growth of S288c [*FLO8*]. These data further corroborate differences in the regulation of *FLO1* and *FLO11*.

The Σ1278*b* yeast strain requires the general control transcription factor of amino acid biosynthesis to activate *FLO11* (Braus *et al*., 2003). The results described above (Fig. 2) already showed that *FLO1* expression of S288c [*FLO8*] is not dependent on the presence or absence of sufficient amount of amino acids. Consistently, the results of Fig. 4 verify that in contrast to Σ1278*b*, which requires *GCN4* for *FLO11* expression, a *gcn4*Δ deletion does not impair haploid adhesive growth under all tested conditions, suggesting that it is not required for *FLO1* expression.

Another transcription factor, Mss11p (van Dyk *et al*., 2005), is essential for adhesive growth of S288c [*FLO8*]. The *mss11*Δ deletion prevented haploid adhesive growth completely under all tested conditions on agar and on plastic (Fig. 4). This impairment cannot be suppressed by overexpression of *FLO8.* The adhesion assay upon glucose limitation on YPD media demonstrates a special role of Mss11p for S288c adherence. The two non-adherent S288c [*FLO8*] derivatives *mss11*Δ and *tpk2*Δ show subtle but significant differences in phenotype upon glucose starvation (Fig. 4C). Whereas the *tpk2*Δ strain still shows a remaining layer of cells on the agar indicating a low level of *FLO11* transcription, the *mss11*Δ cells are entirely washed away indicating repression of both genes, *FLO1* and *FLO11* (Fig. 4C). Therefore, Mss11p is essential for the expression of both adhesin encoding genes.

**Fig. 5:**
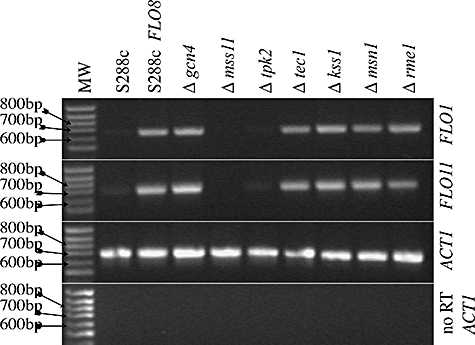
Expression of *FLO1* and *FLO11* transcripts of S288c [*FLO8*] derivatives. *FLO1* and *FLO11* RNA levels of yeast S288c (*flo8*) and S288c [*FLO8*] were compared with transcripts of S288c [*FLO8*] strains carrying deletions in the indicated genes. RNAs were amplified by RT-PCR followed by semiquantitative PCR using *Taq* polymerase. The PCR reactions were separated on 1% TAE-agarose gel electrophoresis. *ACT1* transcripts served as control.

Transcript analyses confirmed the stronger impact of the *mss11*Δ in comparison with the *tpk2*Δ deletion on adherence of S288c [*FLO8*] (Fig. 5). A low but significant *FLO11* expression and no detectable transcript of *FLO1* can be observed in the S288c [*FLO8*] derivative *tpk2*Δ. The total abolishment of adherence in the *mss11*Δ strain correlates with no detectable transcripts of *FLO1* and *FLO11.*

In summary, these data suggest that *FLO1* of S288c [*FLO8*] is primarily under the control of the PKA pathway and the transcription factors Flo8p and Mss11p, whereas other control mechanisms which are known for *FLO11* are missing for activation of *FLO1*.

### Differential regulation and function of *FLO1* and *FLO11* in S288c compared with Σ1278*b* are reflected by differences in the corresponding promoters and coding sequences

The distinct regulations and functions of *FLO1* and *FLO11* in *S. cerevisiae* S288c with an intact regulator Flo8p prompted us to compare these genes to the corresponding genes of strain Σ1278*b*, which also carries an intact Flo8p combined with active *FLO11* (GenBank accession EF670006) but inactive *FLO1* (GenBank accession EF670005)*.* As expected, the sequences of the two active *FLO11* genes of both strains did not show extraordinary differences. In the Σ1278*b FLO11* promoter there are two small insertions at position −407 (TCTTT) and −1976 (AAGAGAATGTCGC) in comparison with S288c. The coding region of Σ1278*b FLO11* shows several single amino acid exchanges spread over the gene, an insertion of 15 amino acids at position 118 and small deletions at several positions (Fig. S1A). Interestingly, the repetitive sequences are differently arranged in Σ1278*b* including five more large and four more short repeats respectively (Fig. S1B). Complementation analyses using Σ1278*b FLO11* revealed complementation of S288c *flo11*Δ and partial complementation of S288c *flo1*Δ in wash tests, whereas the S288c *FLO1* is unable to complement Σ1278*b*Δ*flo11* (data not shown).

**Fig. 6:**
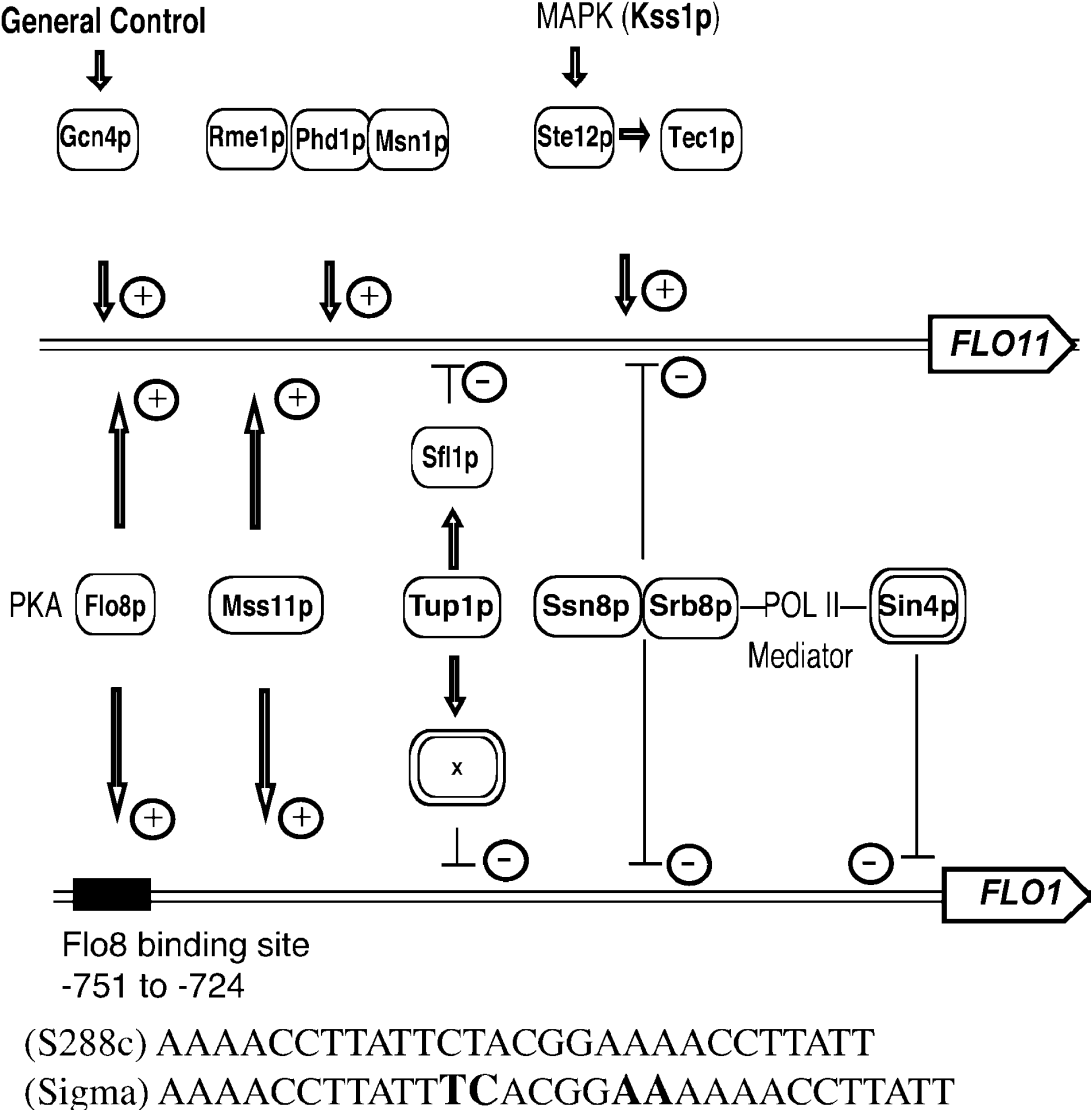
Comparison of the regulation of *FLO11* and *FLO1* transcription of *S. cerevisiae* S288c [*FLO8*]. Shared regulators for the expression of both genes are indicated. The upper box represents additional specific regulation for the more complex *FLO11* promoter; Sin4p is specifically required for *FLO1* expression. Positive regulation is indicated by +, negative regulation by −.

The situation is completely different for the two *FLO1* DNA sequences representing an active gene in S288c [*FLO8*] and an inactive *FLO1* locus in Σ1278*b*. The Flo8 binding site of the *FLO1* promoter in Σ1278*b* is mutated in four positions in comparison with S288c AAAACCTTATTCTACGGAAAACCTTATT at position −751 to −724 of S288c (Kobayashi *et al*., 1999) to AAAACCTTATT**TC**ACGG**AA**AAAACCTTATT (Fig. 6). Furthermore, there are two major deletions from codon 347–526 and codon 680–774 within the *FLO1* open reading frame of Σ1278*b.* These deletions result in the loss of six tandem repeats, which have been shown to be important for adhesion (Verstrepen *et al*., 2005). In addition, we found several minor changes in the downstream part of the gene (Fig. S2). We assume that the change in the Σ1278*b FLO1* promoter and the lack of overall 267 amino acids play a major role that this strain has developed a primarily Flo11p based strategy for adhesion.

## Discussion

The baker's yeast *S. cerevisiae* accompanies human culture in bread making or brewing of alcoholic beverages since several millennia. In addition, yeast strains have been cultivated since many decades after the first isolation of a pure culture which still was showing dimorphism in 1883 by Emil Chr. Hansen (Hansen, 1883) in numerous laboratories and have been selected for non-adherent and non-flocculating phenotypes. A major target of this selection seems to be the *FLO8* transcriptional activator gene, which is mutated in the commonly used *S. cerevisiae* S288c resulting in a completely non-adherent yeast (Liu *et al*., 1996). In this study we analysed the differences in the regulation mechanisms and the distinct functions of the two flocculin encoding genes, *FLO1* and *FLO11*, which can be activated when *FLO8* is restored.

In addition, our data suggest that even *S. cerevisiae*Σ1278*b* has been selected to impair complete Flo8p activator action, because its *FLO1* gene, which cannot be activated, has accumulated mutations in the Flo8p promoter binding site (Fig. 6). Σ1278*b* had been the research object which allowed the rediscovery of dimorphism including adherence and filamentation of *S. cerevisiae* (Gimeno *et al*., 1992), although this phenomenon had been originally described in the 19th century (Hansen, 1883). In this strain the transition from a unicellular to a multicellular organism including the analysis of diploid pseudohyphae and haploid adhesive growth has been primarily studied. Σ1278*b* carries only one expressed *FLO* gene (*FLO11*), which is responsible for all necessary functions within the haploid as well as the diploid yeast life cycle in different environments. The Σ1278*b FLO11* has rearranged and reshaped its repetitive region in comparison with S288c resulting in five more long and four more short repeats (Fig. S1). The repetitive region is important for the efficiency of adhesion and flocculation and the changes might partially reflect the development of a one gene based adhesion strategy (Verstrepen *et al*., 2005). Four other *FLO* genes including *FLO1* are silent in Σ1278b and can only be re-activated by mutation or genomic rearrangements (Verstrepen *et al*., 2004; 2005). The inactive Σ1278b *FLO1*, encoding a protein that is significantly truncated by 267 amino acids when compared with S288c *FLO1* (Fig. S2), might also be a consequence of a genomic reorganization. Another example for the importance of genomic rearrangements for the adaptation of the yeast cell–surface is the foam forming *AWA1* gene product. This protein is present in *S. cerevisiae* strains used for sake production, and is presumably a chimeric protein corresponding to parts of two genes of the commonly used S288c (Alexandre *et al*., 2000; Shimoi *et al*., 2002). Accordingly S288c is unable to form foams or biofilms, even after the restauration of *FLO8* (Fig. 1A).

Cell–surface diversity, which can be the key to virulence in a host–pathogen relationship, is primarily the result of differentially expressed genes for surface proteins. An additional level of adaptation to the environment can be mediated by stochastic processes that result in a variegated expression of surface proteins in an otherwise homogeneous population (Halme *et al*., 2004). In naturally fluctuating environments stochastic switching patterns might be more effective than sensing mechanisms (Kussell and Leibler, 2005). The diversity of cell–surfaces in pathogen populations is an important strategy to become less accessible for hosts. Many organisms have developed strategies to model their appearance by recombining, silencing or activating different genes for their cell–surface proteins (Esser and Schoenbechler, 1985; Kyes *et al*., 2001). Pathogenic yeasts such as *Candida albicans* or *Candida glabrata* express multiple agglutinin-like *ALS* (Hoyer, 2001) and epithelial adhesion *EPA* genes (De Las Penas *et al*., 2003) respectively. The three morphological *Candida* forms, single cell yeasts, pseudohyphae and true hyphae, significantly differ in their cell–surface and are also partially regulated by Flo8p (Lopez-Ribot, 2005; Nobile and Mitchell, 2005; Ramage *et al*., 2005). *S. cerevisiae* represents a yeast with a significant less complex arsenal of variant surface proteins compared with *Candida*. It consists of only two active genes of the five members of the *FLO* family in *FLO8*-restored strain S288c, which has been used in this study. However, a limited number of flocculin genes have already enough potential to cause a significant impact on different habitats. One example is the *S. cerevisiae* outbreak of fungemia among intensive care unit patients that has been reported (Cassone *et al*., 2003). In S288c [*FLO8*] the two flocculin genes *FLO1* and *FLO11* are kept in a metastable state and can be either silenced and repressed or activated differentially. The activator Flo8p which cooperates with other factors like Mga1p and Mss11p (Bester *et al*., 2006; Borneman *et al*., 2006) is able to activate both genes, but is only one of many genes and their products which are involved in the control of *FLO1* and *FLO11*.

The molecular mechanisms controlling *FLO1* and *FLO11* share similarities as well as significant differences (Fig. 6). Repression and/or silencing of *FLO1* as well as of *FLO11* depends on an intact mediator complex of the RNA polymerase II, which has different functions including chromatin remodelling. *FLO1* and *FLO11* repression requires Srp8p and Ssn8p, both components of the Cdk8 mediator subcomplex. It is yet unknown whether there is a connection between Srp8p and Ssn8p and the epigenetic control of *FLO* silencing by different histone-deacetylases as it has been described (Halme *et al*., 2004). Cdk8 is also involved in phosphorylation of the RNA–polymerase C-terminal-domain and therefore is part of the transcriptional repression control (Liao *et al*., 1995; Holstege *et al*., 1998). In addition, Cdk8 phosphorylates and therefore destabilizes Ste12p or Gcn4p which are transcriptional activators of *FLO11* (Chi *et al*., 2001; Nelson *et al*., 2003). It is yet unclear which of these functions is necessary to turn off the two *FLO* genes. In addition to the common function of the mediator components Srb8p and Ssn8p in repressing *FLO1* and *FLO11*, there is an additional specific function of Sin4p, which is only required for the repression of *FLO1* but not for *FLO11*. Sin4p is part of the mediator tail interacting with various transcriptional activators (Pan and Heitman, 1999; Park *et al*., 2000), and it remains to be elucidated which interactions are important for the specific effect of Sin4p on *FLO1*. Sfl1p, the key repressor of *FLO11*, shares its promoter binding site with the Flo8p activator (Pan and Heitman, 2002). However, the finding that Sfl1p is not required for *FLO1* repression represents another important difference in the regulation of both *FLO* genes. Tup1p, the corepressor of Sfl1p in the *FLO11* promoter (Conlan and Tzamarias, 2001), is known as a corepressor for different DNA-binding proteins and is also required for *FLO1* repression. It remains to be elucidated which protein is the partner in *FLO1* repression. The *FLO1* and *FLO11* expression depends essentially on the Mss11p transcription factor (van Dyk *et al*., 2005). A deletion of *MSS11* can be suppressed neither by overexpression of activators nor by deletion of repressors. This suggests Mss11p as being a key player for yeast cell–cell and cell–surface interactions. *FLO1* and *FLO11* are regulated by the cAMP–PKA cascade including the Tpk2p catalytic subunit of PKA (Mösch *et al*., 1999; Cullen and Sprague, 2000; Sengupta *et al*., 2007) and the transcription factor Flo8p. Other regulators of *FLO11* (Fig. 6) including the MAPK (*KSS1*) pathway are not relevant for *FLO1* expression, suggesting significant differences in the regulation mechanism of both genes.

The two active *FLO* genes of S288c carrying a restored *FLO8* correspond to only partially overlapping and mostly distinct functions. *FLO1* is primarily important for haploids and allows cell–cell interactions, which corresponds to the finding that it is expressed in several industrial yeasts (Kobayashi *et al*., 1996; 1998; 1999). In industry, flocculation can be a desirable property, allowing easy separation of products and biomass. Flo1p also supports cell–substrate interactions of haploids under specific environmental conditions. The other active *FLO* gene of S288c, *FLO11*, encodes the typical surface marker of diploids. The carefully regulated *FLO11* is essential for the developmental programme, which results in the formation of diploid pseudohyphae with their distinct features (Kron *et al*., 1994). Interestingly, both S288c *FLO* genes are required for an appropriate morphological response in a specific environment. An example of such a response is the growth of haploid S288c cells on a surface during glucose starvation. Flo11p functions as a first adhesin to establish the initial cell–substrate interaction of the first layer of cells. Flo1p is responsible for the second step and increases the population at this specific spot of the habitat by adding additional layers of cells due to its cell–cell interaction potential.

Our studies have revealed several differences in the regulation and function of the two Flo8p regulated genes, *FLO1* and *FLO11*, of the budding yeast. It will be interesting to analyse in the future, if natural *S. cerevisiae* wild-type yeasts even express a still larger portion of the *FLO* gene reservoir and what additional surface protein encoding genes have been created by reshaping the genome for additional functions in specific environmental conditions.

## Experimental procedures

### General methods, yeast strains and plasmids

Cultivations of *S. cerevisiae* in SC or rich YPD media and yeast methods including genetic crosses and transformation were carried out as described previously (Gietz *et al.*, 1992; Sherman, 1991). The yeast strains used in this study (Table 2) are either derivatives of *S. cerevisiae* strain Σ1278b also known as MB1000 and MB758-5b (Brandriss and Magasanik, 1979; Siddiqui and Brandriss, 1988), or of the S288c strain-derived BY-series (Brachmann *et al*., 1998). Deletions of *FLO1* and *FLO11* in strains Y06870 (*flo1*Δ) and Y05953 (*flo11*Δ) were confirmed by PCR. Plasmids of the YCplac and YEplac series used for complementation of auxotrophic marker alleles were described previously (Gietz and Sugino, 1988). *FLO8* carrying plasmids pHL1 (*ARS-CEN*, low copy), pHL11 (integrative) and pHL135 (2 μ, high copy) which were used for complementation of S288c *flo8*, were described previously (Liu *et al*., 1996). Amino acid starvation was induced by the histidine analogue 3AT. Strains used for 3AT adhesion tests were reversed to histidine prototrophy by integrating a *HIS3* 1.7 kb BamHI fragment from pBR322-Sc2676 (Struhl and Davis, 1980) at its original locus.

**Table 2:** *S. cerevisiae* strains used in this study.

Yeast strain	Genotype	Background	Source
BY4741 (WT)	*MATa*; *his3*Δ*1*; *leu2*Δ*0*; *met15*Δ*0*; *ura3*Δ*0*	S288c	Euroscarf
BY4742 (WT)	*MATα*; *his3*Δ*1*; *leu2*Δ*0*; *lys2*Δ*0*; *ura3*Δ*0*	S288c	Euroscarf
BY4743 (WT)	*MATa/α*; *his3*Δ*1*/*his3*Δ*1*; *leu2*Δ*0*/*leu2*Δ*0*; *met15*Δ*0*/*MET15*; *LYS2*/*lys2*Δ*0*; *ura3*Δ*0*/*ura3*Δ*0*	S288c	Euroscarf
Y05351 (*ssn8*Δ)	Like BY4741; but *YNL025c*::*kanMX4*	S288c	Euroscarf
Y01976 (*sin4*Δ)	Like BY4741; but *YNL236w*::*kanMX4*	S288c	Euroscarf
Y04296 (*mrpl28*Δ)	Like BY4741; but *YDR462w*::*kanMX4*	S288c	Euroscarf
Y05799 (*srb8*Δ)	Like BY4741; but *YCR081w*::*kanMX4*	S288c	Euroscarf
Y07198 (*tup1*Δ)	Like BY4741; but *YCR084c*::*kanMX4*	S288c	Euroscarf
Y02396 (*sfl1*Δ)	Like BY4741; but *YOR140w*::*kanMX4*	S288c	Euroscarf
Y06870 (*flo1*Δ)	Like BY4741; but *YAR050w*::*kanMX4*	S288c	Euroscarf
Y07106 (*flo10*Δ)	Like BY4741; but *YKR102w*::*kanMX4*	S288c	Euroscarf
Y05953 (*flo11*Δ)	Like BY4741; but *YIR019c*::*kanMX4*	S288c	Euroscarf
Y00249 (*gcn4*Δ)	Like BY4741; but *YEL009c*::*kanMX4*	S288c	Euroscarf
Y07155 (*tec1*Δ)	Like BY4741; but *YBR083w*::*kanMX4*	S288c	Euroscarf
Y01089 (*tpk2*Δ)	Like BY4741; but *YPL203w*::*kanMX4*	S288c	Euroscarf
Y04674 (*rem1*Δ)	Like BY4741; but *YGR044c*::*kanMX4*	S288c	Euroscarf
Y06981 (*kss1*Δ)	Like BY4741; but *YGR040w*::*kanMX4*	S288c	Euroscarf
Y06266 (*msn1*Δ)	Like BY4741; but *YOL116w*::*kanMX4*	S288c	Euroscarf
Y00747 (*mss11*Δ)	Like BY4741; but *YMR164c*::*kanMX4*	S288c	Euroscarf
Y16870 (*flo1*Δ)	Like BY4742; but *YAR050w*::*kanMX4*	S288c	Euroscarf
Y17106 (*flo10*Δ)	Like BY4742; but *YKR102w*::*kanMX4*	S288c	Euroscarf
Y15953 (*flo11*Δ)	Like BY4742; but *YIR019c*::*kanMX4*	S288c	Euroscarf
Y35953 (*flo11*Δ)	Like BY4743; but *YIR019c*::*kanMX4*/*YIR019c*::*kanMX4*	S288c	Euroscarf
Y37106 (*flo11*Δ)	Like BY4743; but *YKR102w*::*kanMX4*/*YKR102w*::*kanMX4*	S288c	Euroscarf
Y36870 (*flo1*Δ)	Like BY4743; but *YAR050w*::*kanMX4*/*YAR050w*::*kanMX4*	S288c	Euroscarf
RH2848 (WT)	*MATa*; *ura3-52*; *leu2*::*hisG*	Σ1278*b*	Braus *et al*. (2003)
RH2662 (*flo11*Δ)	*MATa*; *ura3-52*; *flo11*::*kanMX4*	Σ1278*b*	Braus *et al*. (2003)
RH2652 (*flo8*Δ)	*MATa*; *ura3-52*; *flo8*::*kanMX4*	Σ1278*b*	Braus *et al*. 2003)
RH2656 (WT)	*MATa/α*	Σ1278*b*	Braus *et al*. (2003)
RH3276 (*flo1*Δ; *flo11*Δ)	Like BY4741; but *YAR050w*::*kanMX4*; *YIR019c*::*kanMX4*	S288c	This study

### Growth tests

All strains used in the different growth tests were either diploid (pseudohyphae) or *MATa.* Tests for adhesive growth on agar were performed as described (Roberts and Fink, 1994; Braus *et al*., 2003). Adhesion to plastic surface was assayed in 96 well flat bottom plates (Reynolds and Fink, 2001). Pseudohyphal growth was assayed on synthetic low ammonia dextrose (SLAD) medium (Gimeno and Fink, 1992) using diploid strains. For assaying flocculation, yeast strains were grown overnight in SC media and their ability to flocculate was judged from the presence of visible cell aggregates in the media and quantified as described before (Kobayashi *et al*., 1996) using the equation FA = 1 − B/A, where A is OD_600_ in the absence and B in the presence of 0.1% CaCl_2_. To assay the formation of biofilms, mat formation was monitored on YPD plates with 0.3% (w/v) agar and 0.2% glucose (Reynolds and Fink, 2001) and photographed after 4 days of incubation at 25°C.

### Screening yeast deletion collection

We performed *flo8* suppressor screening using a Freedom Evo robot (TECAN) and the systematic *MATa* yeast deletion collection (Brachmann *et al*., 1998). The 4895 individual collection deletion strains were grown in 96 well microtiter plates in liquid SC media for 24 h. Cells were stained by crystal violet and non-adhesive cells were washed off the plates (Reynolds and Fink, 2001). Wells containing adhesive deletion strains were further analysed.

### Gene transcription analyses

Cells were grown in SC media to an OD_600_ of 0.7, total RNA was isolated (Cross and Tinkelenberg, 1991) and samples were treated afterwards with RNase free DNase (Qiagen). RT-PCR experiments involved equal amounts of total RNA (1 μg) subjected to first-strand cDNA synthesis with the Reverse Aid™ kit (MBI Fermentas) according to the manufacturer's recommendations. After first-strand syntheses, 1/20 of the cDNA was used for semiquantitative PCR (Frohloff *et al*., 2001). Oligonucleotide primers used for specific amplification were as follows: *FLO1* (RT1A: 5′-CTCATCGCTATATGTTTTTGG-3′, RT1B: 5′-CGAGTAAACAACCTTCATTGG-3′), *FLO11* (RT11A: 5′-CATTTCTACTCGCTTATTTGG-3′, RT11B: 5′-CGGAAGTGCTAGATGTAGTGG-3′), *ACT1* (RTactA: 5′-ATTCTGAGGTTGCTGCTTTGG-3′, RTactB: 5′-GAAGATTGAGCAGCGGTTTGC-3′), *FLO5* (RT5A: 5′-CCCCAACAAACGTAACCC-3′, RT5B: 5′-GTTGACCGTTGGTACCGG-3′), *FLO9* (RT9A: 5′-CTACCATAACTACAACGG-3′, RT9B: 5′-GCAAACCATTGGTACCGG-3′), *FLO10* (RT10A: 5′-GAGTTCAGATCTGTTCGG-3′, RT10B: 5′-CCGTACAGACTTCACTGG-3′). The specific annealing of *FLO1* and *FLO11* primers has been checked by PCR on chromosomal DNA of the respective deletion strains.

## Supplementary material

This material is available as part of the online article from: http://www.blackwell-synergy.com/doi/abs/10.1111/j.1365-2958.2007.06014.x

(This link will take you to the article abstract).
