# Supplementary material for: Differential Flo8p-dependent regulation of FLO1 and FLO11 for cell–cell and cell–substrate adherence of S. cerevisiae S288c
Source: Mol Microbiol. 2007 Dec;66(5):1276–89. doi: 10.1111/j.1365-2958.2007.06014.x (PMC2780560; doi:10.1111/j.1365-2958.2007.06014.x)
Supplement: Supplementary file 1 [file mmi0066-1276-SD1.pdf]

Supplementary material

Sequence analysis of the *S. cerevisiae* Σ1278*b FLO11* (Figure S1) and *FLO1* (Figure S2) coding region revealed several differences including single amino acid exchanges, deletions and insertions when compared to the respective S288c sequences. In addition, the repetitive regions of Σ1278b *FLO11* have been rearranged and enlarged in comparison to S288c (Figure S1B). Two major deletions at amino acids 374 to 554 and amino acids 681 to 770 shorten the repetitive sequence region of Σ1278*b* Flo1p and result in a significantly truncated product (Figure S2).

Figure S1. Comparison of *S. cerevisiae* S288c and Σ1278*b* Flo11p amino acid sequence. Differences are indicated in blue and black. (A) Alignment of the two sequences. (B+C) Comparison of the S288c (B) and Σ1278*b* (C) *FLO11* repetitive sequence regions. Different nucleotides are shown in different colors. The S288c *FLO11* repetitive sequence region consists of 24 long and 16 short repeats. The Σ1278*b FLO11* repetitive sequence region consists of 29 long and 20 short repeats. The arrangement of long and short repeats varies between the *FLO11* genes of both strains.

Figure S2. Comparison of *S. cerevisiae* S288c and Σ1278*b* Flo1p amino acid sequence. Differences are indicated in blue and black.

```
           1         10         20         30         40         50         60         70         80         90        100        110        120        130
Flo11S288c MQRPFLLAYLVLSLLFNSALGFPTALVPRGSSEGTSCNSIVNGCPNLDFNWHMDQQNIMQYTLDVTSVSWVQDNTYQITIHVKGKENIDLKYLWSLKIIGVTGPKGTVQLYGYNENT------------
Flo11Sigma MQRPFPFAYLVLSLLFYSALGFPTALVPRGSSEGTSCNSIVNGCPNLDFNWHMNQQNIMQYTLDVTSVSWVQDNTYQITIHVKGEENIDLKYLSSLKIIGVTGPKDTVQLYGYDENTDWIDNPLVSRCDE
Consensus  MQRPFllAYLVLSLLFnSALGFPTALVPRGSSEGTSCNSIVNGCPNLDFNWHM#QQNIMQYTLDVTSVSWVQDNTYQITIHVKGeENIDLKYLsSLKIIGVTGPKdTVQLYGY#ENT............

           131  140        150        160        170        180        190        200        210        220        230        240        250        260
Flo11S288c --YLIDNPTDFTATFEVYATQDVNSCQVWMPNFQIQFEYLQGSAAQYASSWQWGTTSFDLSTGCNNYDNQGHSQTDFPGFYWNIDCDNNCGGTKSSTTTSSTSESSTTTSSTSESSTTTSSTSESSTTTS
Flo11Sigma NTYLIDNPTDFTATFEVYATQDVNSCQVWMPNFQIQFEYLQGSAAEYACSWEWGTTSFYLSTGCDNYDNQGYSQTDFPGFYWNIDCDNNCAPVP-------TPSSSTTESSSAPVPTPSSSTTESSSAPA
Consensus  ..YLIDNPTDFTATFEVYATQDVNSCQVWMPNFQIQFEYLQGSAA#YAcSW#WGTTSFdLSTGC#NYDNQGhSQTDFPGFYWNIDCDNNCagtk.......TpeSSTTeSSsaespTpsSSTsESSsapa

           261  270        280        290        300        310        320        330        340        350        360        370        380        390
Flo11S288c STSESSTSSTTAPATPTTTSCTKEKPTPPTTTSCTKEKPTPPHHDTTPCTKKKTTTSKTCTKKTTTPVPTPSSSTTESSSAPVPTPSSSTTESSSAPVTSSTTESSSAPVPTPSSSTTESSSAPVTSST
Flo11Sigma PTPSSSTTESSSAPA-PTPSSSTTESSSAPVTSSTTES-------------------------SSAPAPTPSSSTTESSSAPAPTPSSSTTESSSAPVTSSTTESSSAPV---TSSTTESSSAPVTSST
Consensus  pTpeSSTseSssAPA.PTpsScTkEkpsaPtTsScTee.........................ssaPaPTPSSSTTESSSAPaPTPSSSTTESSSAPVTSSTTESSSAPV...sSSTTESSSAPVTSST

           391  400        410        420        430        440        450        460        470        480        490        500        510        520
Flo11S288c TESSSAPV---TSSTTESSSAPVPTPSSSTTESSSAPVTSSTTESSSAPVTSSTTESSSAPVTSSTTESSSAPVT---SSTTESSSAPVPTPSSSTTESSSAPVTSSTTESSSAPVPTPSSSTTESSSAP
Flo11Sigma TESSSAPAPTPSSSTTESSSAPAPTPSSSTTESSSAPVTSSTTESSSAPVTSSTTESSSAPVTSSTTESSSAPVPTPCSSTTESSSAPAPTPSSSTTESSSAPATSSTTESSSAPVPTPSSSTTESSSAP
Consensus  TESSSAPa...sSSTTESSSAPaPTPSSSTTESSSAPVTSSTTESSSAPVTSSTTESSSAPVTSSTTESSSAPVp...SSTTESSSAPaPTPSSSTTESSSAPaTSSTTESSSAPVPTPSSSTTESSSAP

           521  530        540        550        560        570        580        590        600        610        620        630        640        650
Flo11S288c ---------------VTSSTTESSSAPVPTPSSSTTESSSAPAPTPSSSTTESSSAPVTSSTTESSSAPVPTPSSSTTESSSTPVTSSTTESSSAPVPTPSSSTTESSSAPVPTPSSSTTESSSAPAPTP
Flo11Sigma APTPSSSTTESSSAPVTSSTTESSSAPAPTPSSSTTESSSAPAPAPSSSTTESSSAPVTSSTTESSSAPV---TSSTTESSSAPVTSSTTESSSAPAPTPSSSTTESSSAPAPTPSSSTTESSSAPV---
Consensus  ...............VTSSTTESSSAPaPTPSSSTTESSSAPAPaPSSSTTESSSAPVTSSTTESSSAPV...sSSTTESSSaPVTSSTTESSSAPaPTPSSSTTESSSAPaPTPSSSTTESSSAPa...

           651  660        670        680        690        700        710        720        730        740        750        760        770        780
Flo11S288c SSSTTESSSAPVTSSTTESSSAPVPTPSSSTTESSSAPVPTPSSSTTESSSAPVPTPSSSTTESSSAPVTSSTTESSSAPV---TSSTTESSSAPVPTPSSSTTESSSAPVPTPSSSTTESSSAPVPTPS
Flo11Sigma TSSTTESSSAPVTSSTTESSSAPV---TSSTTESSSAPVPTPCSSTTESSSAPAPTPSSSTTESSSAPATSSTTESSSAPVPTPSSSTTESSSAPAPTPSSSTTESSSAPV---TSSTTESSSAPVPTPS
Consensus  sSSTTESSSAPVTSSTTESSSAPV...sSSTTESSSAPVPTPcSSTTESSSAPaPTPSSSTTESSSAPaTSSTTESSSAPV...sSSTTESSSAPaPTPSSSTTESSSAPV...sSSTTESSSAPVPTPS

           781  790        800        810        820        830        840        850        860        870        880        890        900        910
Flo11S288c SSTTESSSAPVT---SSTTESSSAPVPTPSSSTTESSSAPVPTPSSSTTESSSAPVPTPSSSTTESSVAPVPTPSSSSNITSSAPSSTPFSSSTESSSVPVPTPSSSTTESSSAP---------------
Flo11Sigma SSTTESSSAPVPTPCSSTTESSSAPVPTPSSSTTESSSAPVPTPSSSTTESSSAPV---TSSTTESSVAPVPTPSSSSNITSSAPSSTPFSSSTESSSVPVPTPSSSTTESSSAPAPTPSSSTTESSSAP
Consensus  SSTTESSSAPVp...SSTTESSSAPVPTPSSSTTESSSAPVPTPSSSTTESSSAPV...sSSTTESSVAPVPTPSSSSNITSSAPSSTPFSSSTESSSVPVPTPSSSTTESSSAP...............

           911  920        930        940        950        960        970        980        990       1000       1010       1020       1030       1040
Flo11S288c VSSSTTESSVAPVPTPSSSSNITSSAPSSIPFSSTTESFSTGTTVTPSSSKYPGSQTETSVSSTTETTIVPTKTTTSVTTPSTTTITTTVCSTGTNSAGETTSGCSPKTVTTTVPTTTTTSVTTSSTTTI
Flo11Sigma VSSSTTESSVAPVPTPSSSSNITSSAPSSTPFSSSTESSSVPVP-TPSSSTTESSSAPVS-SSTTESSVAPVPTPSS-----SSNITSSAPSSSKYPGSQTETSVSSTTETTIVPTTTTTSVTTPSTTTI
Consensus  VSSSTTESSVAPVPTPSSSSNITSSAPSSiPFSSsTESfStgtp.TPSSSktegSqaetS.SSTTEss!aPtkTpsS.....ssnITssacSsgknpag#TesgcSpkTeTTiVPTTTTTSVTTpSTTTI

           1041 1050       1060       1070       1080       1090       1100       1110       1120       1130       1140       1150       1160       1170
Flo11S288c TTTVCSTGTNSAGETTSGCSPKTITTTVPCSTSPSETASESTTTSPTTPVTTVVSTTVVTTEYSTSTKPGGEITTTFVTKNIPTTYLTTIAPTPSVTTVTNFTPTTITTTVCSTGTNSAGETTSGCSPKT
Flo11Sigma TTTVCSTGTNSAGETTSGCSPKTITTTVPCSTSPSETASESTTTSPTTPVTTVVSTTVVTTEYSTSTKPGGEITTTFVTKNIPTTYLTTIAPTPSVTTVTNFTPTTITTTVCSTGTNSAGETTSGCSPKT
Consensus  TTTVCSTGTNSAGETTSGCSPKTITTTVPCSTSPSETASESTTTSPTTPVTTVVSTTVVTTEYSTSTKPGGEITTTFVTKNIPTTYLTTIAPTPSVTTVTNFTPTTITTTVCSTGTNSAGETTSGCSPKT

           1171 1180       1190       1200       1210       1220       1230       1240       1250       1260       1270       1280       1290       1300
Flo11S288c VTTTVPCSTGTGEYTTEATTLVTTAVTTTVVTTESSTGTNSAGKTTTGYTTKSVPTTYVTTLAPSAPVTPATNAVPTTITTTECSAATNAAGETTSVCSAKTIVSSASAGENTAPSATTPVTTAIPTTVI
Flo11Sigma VTTTVPCSTGTGEYTTEATTPVTTAVTTTVVTTESSTGTNSAGETTTGYTTKSVPTTYVTTLAPSAPVTPATSAVPTTITTTECSAATNAAGETTSVCSAKTIVSSASAGENT-----TPVTTAIPTTVV
Consensus  VTTTVPCSTGTGEYTTEATTlVTTAVTTTVVTTESSTGTNSAGeTTTGYTTKSVPTTYVTTLAPSAPVTPATnAVPTTITTTECSAATNAAGETTSVCSAKTIVSSASAGENT.....TPVTTAIPTTV!

           1301 1310       1320       1330       1340       1350       1360       1370       1380       1390       1400       1410       14201424
Flo11S288c TTESSVGTNSAGETTTGYTTKSIPTTYITTLIPGSNGAKNYETVATATNPISIKTTSQLATTASASSVAPVVTSPSLTGPLQSASGSAVATYSVPSISSTYQGAANIKVLGNFMWLLLALPVVF
Flo11Sigma TTESSVGTNSAGETTTGYTTKSIPTTYITTLIPGSNGAKNYETVATATNPISIKTTSQLATTASASSMAPVVTSPSLTGPLQSASGSAVATYSVPSISSTYQGAANIKVLGNFMWLLLALPVVF
Consensus  TTESSVGTNSAGETTTGYTTKSIPTTYITTLIPGSNGAKNYETVATATNPISIKTTSQLATTASASSmAPVVTSPSLTGPLQSASGSAVATYSVPSISSTYQGAANIKVLGNFMWLLLALPVVF
```

Figure S1 A

B

FLO11(S288c)

```
 1 GACTACTACTCCAGTACCAACCCCATCAAGCTCTACTACTGAAAG
 2 TTCTTCTGCTCCAGTACCAACTCCATCCAGCTCTACTACTGAAAG
 3 CTCTTCTGCTCCAG         TAACCAGCTCTACCACTGAAAG
 4 CTCTTCTGCTCCAGTACCAACCCCATCAAGCTCTACTACTGAAAG
 5 CTCTTCTGCTCCAG         TAACCAGCTCTACCACTGAAAG
 6 CTCTTCTGCTCCAG         TAACCAGCTCTACCACTGAAAG
 7 CTCTTCTGCTCCAGTACCAACTCCATCCAGCTCTACTACTGAAAG
 8 CTCTTCTGCTCCAG         TAACCAGCTCTACCACTGAAAG
 9 CTCTTCTGCTCCAG         TAACCAGCTCTACCACTGAAAG
10 CTCTTCTGCTCCAG         TAACCAGCTCTACCACTGAAAG
11 CTCTTCTGCTCCAG         TAACCAGCTCTACCACTGAAAG
12 CTCTTCTGCTCCAGTACCAACTCCATCCAGCTCTACTACTGAAAG
13 CTCTTCTGCTCCAG         TAACCAGCTCTACCACTGAAAG
14 CTCTTCTGCTCCAGTACCAACTCCATCCAGCTCTACTACTGAAAG
15 CTCTTCTGCTCCAG         TAACCAGCTCTACCACTGAAAG
16 CTCTTCTGCTCCAGTACCAACTCCATCCAGCTCTACTACTGAAAG
17 CTCTTCTGCTCCAGCTCCAACCCCATCAAGCTCCACCACTGAAAG
18 CTCTTCTGCTCCAG         TAACCAGCTCTACCACTGAAAG
19 CTCTTCTGCTCCAGTACCAACTCCATCCAGCTCTACTACTGAAAG
20 CTCTTCTACTCCAG         TAACCAGCTCCACCACTGAAAG
21 CTCTTCTGCTCCAGTGCCAACTCCATCAAGCTCTACTACTGAAAG
22 CTCTTCTGCTCCAGTACCAACTCCATCAAGCTCTACTACTGAAAG
23 CTCTTCTGCTCCAGCTCCAACCCCATCAAGCTCTACTACTGAAAG
24 CTCTTCTGCTCCAG         TAACCAGCTCTACCACTGAAAG
25 CTCTTCTGCTCCAGTACCAACTCCATCCAGCTCTACTACTGAAAG
26 CTCTTCTGCTCCAGTACCAACCCCATCCAGCTCTACTACTGAAAG
27 CTCTTCTGCTCCAGTACCAACTCCATCCAGCTCTACTACTGAAAG
28 CTCTTCTGCTCCAG         TAACCAGCTCTACCACTGAAAG
29 CTCTTCTGCTCCAG         TAACCAGCTCTACCACTGAAAG
30 CTCTTCTGCTCCAGTACCAACTCCATCCAGCTCTACTACTGAAAG
31 CTCTTCTGCTCCAGTACCAACTCCATCCAGCTCTACTACTGAAAG
32 CTCTTCTGCTCCAGTACCAACTCCATCCAGCTCTACTACTGAAAG
33 CTCTTCTGCTCCAG         TAACCAGCTCTACCACTGAAAG
34 CTCTTCTGCTCCAGTACCAACTCCATCCAGCTCTACTACTGAAAG
35 CTCTTCTGCTCCAGTGCCAACTCCATCCAGCTCTACTACCGAAAG
36 CTCTTCTGCTCCAGTACCAACCCCATCAAGCTCTACTACTGAAAG
37 CTCTGTAGCACCAGTACCAACCCCATCTTCCTCTAGCAACATCACTTCCTCCGCTCCATCTTCAACCCCATTCAGCTCTAGCACTGAAAG
38 CTCTTCTGTTCCAGTACCAACCCCATCAAGCTCTACTACTGAAAG
39 CTCTTCTGCTCCAG         TATCCAGCTCCACCACTGAAAG
40 CTCTGTAGCACCAGTACCAACCCCATCTTCCTCTAGCAACATCACTTCCTCCGCTCCATCTTCAATTCCATTCAGCTCTACTACTGAAAG

   CTTTTCTACTGGCACTACTGTCACTCCATCATCATCCAAATACCCTGGCAGTCAAACAGAAACCTCTGTTTCTTCTACAACCGAAACTAC
```

C

FLO11(Sigma1278b)

```
 1 CAATTGTGCTCCAGTACCAACTCCATCCAGCTCTACTACTGAAAG
 2 CTCTTCTGCTCCAGTACCAACTCCATCAAGCTCTACTACTGAAAG
 3 CTCTTCTGCTCCAGCTCCAACTCCATCCAGCTCTACTACCGAAAG
 4 CTCTTCTGCTCCAGCTCCAACTCCATCCAGCTCTACCACTGAAAG
 5 CTCTTCTGCTCCAG         TAACCAGCTCTACCACTGAAAG
 6 CTCTTCTGCTCCAGCTCCAACTCCATCCAGCTCTACTACTGAAAG
 7 CTCTTCTGCTCCAGCTCCAACTCCATCCAGCTCTACCACTGAAAG
 8 CTCTTCTGCTCCAG         TAACCAGCTCTACCACTGAAAG
 9 CTCTTCTGCTCCAG         TAACCAGCTCTACCACTGAAAG
10 CTCTTCTGCTCCAG         TAACCAGCTCTACCACTGAAAG
11 CTCTTCTGCTCCAGCTCCAACTCCATCCAGCTCTACTACTGAAAG
12 CTCTTCTGCTCCAGCTCCAACTCCATCCAGCTCTACCACTGAAAG
13 CTCTTCTGCTCCAG         TAACCAGCTCTACCACTGAAAG
14 CTCTTCTGCTCCAG         TAACCAGCTCTACCACTGAAAG
15 CTCTTCTGCTCCAG         TAACCAGCTCTACCACTGAAAG
16 CTCTTCTGCTCCAGTACCAACTCCATGCAGCTCTACTACTGAAAG
17 CTCTTCTGCTCCAGCTCCAACTCCATCCAGCTCTACTACCGAAAG
18 CTCTTCTGCTCCAG         CAACCAGCTCTACCACTGAAAG
19 CTCTTCTGCTCCAGTACCAACTCCATCAAGCTCTACTACTGAAAG
20 CTCTTCTGCTCCAGCTCCAACTCCATCCAGCTCTACTACCGAAAG
21 CTCTTCTGCTCCAG         TAACCAGCTCTACCACTGAAAG
22 CTCTTCTGCTCCAGCTCCAACTCCATCCAGCTCTACTACTGAAAG
23 CTCTTCTGCTCCAGCTCCAGCTCCATCCAGCTCTACCACTGAAAG
24 CTCTTCTGCTCCAG         TAACCAGCTCTACCACTGAAAG
25 CTCTTCTGCTCCAG         TAACCAGCTCTACCACTGAAAG
26 CTCTTCTGCTCCAG         TAACCAGCTCTACCACTGAAAG
27 CTCTTCTGCTCCAGCTCCAACTCCATCCAGCTCTACTACTGAAAG
28 CTCTTCTGCTCCAGCTCCAACTCCATCCAGCTCTACCACTGAAAG
29 CTCTTCTGCTCCAG         TAACCAGCTCTACCACTGAAAG
30 CTCTTCTGCTCCAG         TAACCAGCTCTACCACTGAAAG
31 CTCTTCTGCTCCAG         TAACCAGCTCTACCACTGAAAG
32 CTCTTCTGCTCCAGTACCAACTCCATGCAGCTCTACTACTGAAAG
33 CTCTTCTGCTCCAGCTCCAACTCCATCCAGCTCTACTACCGAAAG
34 CTCTTCTGCTCCAG         CAACCAGCTCTACCACTGAAAG
35 CTCTTCTGCTCCAGTACCAACTCCGTCAAGCTCTACTACTGAAAG
36 CTCTTCTGCTCCAGCTCCAACTCCATCCAGCTCTACTACCGAAAG
37 CTCTTCTGCTCCAG         TAACCAGCTCTACCACTGAAAG
38 CTCTTCTGCTCCAGTACCAACTCCATCCAGCTCTACCACTGAAAG
39 CTCTTCTGCTCCAGTACCAACTCCATGCAGCTCTACTACTGAAAG
40 CTCTTCTGCTCCAGTACCAACTCCATCAAGCTCTACTACTGAAAG
41 CTCTTCTGCTCCAGTACCAACTCCATCAAGCTCTACTACTGAAAG
42 CTCTTCTGCTCCAG         TAACCAGCTCCACCACTGAAAG
43 CTCTGTAGCACCAGTACCAACCCCATCTTCCTCTAGCAACATCACTTCCTCCGCTCCATCTTCAACTCCATTCAGCTCTAGCACTGAAAG
44 CTCTTCTGTTCCAGTACCAACTCCATCCAGCTCTACTACCGAAAG
45 CTCTTCTGCTCCAGCTCCAACTCCATCCAGCTCTACTACTGAAAG
46 CTCCTCTGCTCCAG         TATCCAGCTCCACCACTGAAAG
47 CTCTGTAGCACCAGTACCAACCCCATCTTCCTCTAGCAACATCACTTCCTCGGCTCCATCTTCAACTCCATTCAGCTCTAGCACTGAAAG
48 CTCTTCTGTTCCAGTACCAACTCCATCCAGCTCTACTACTGAAAG
49 CTCCTCTGCTCCAG         TATCCAGCTCCACCACTGAAAG

   CTCTGTAGCACCAGTACCAACCCCATCTTCCTCTAGCAACATCACTTCCTCCGCTCCATCATCATCCAAATACCCTGGCAGTCAAACAGA
```

Figure S1 B+C

```
           1        10        20        30        40        50        60        70        80        90        100       110       120      130
           |--------+---------+---------+---------+---------+---------+---------+---------+---------+---------+---------+---------+---------|
Flo1S288C  MTMPHRYMFLAVFTLLALTSVASGATEACLPAGQRKSGMNINFYQYSLKDSSTYSNAAYMAYGYASKTKLGSVGGQTDISIDYNIPCVSSSGTFPCPQEDSYGNWGCKGMGACSNSQGIAYWSTDLFGFY
Flo1Sigma  MTMPHRYMFLAVFTLLALINVASGATEACLPAGQRKSGMNINFYQYSLKDSSTYSNAAYMAYGYASKTKLGSVGGQTDISIDYNIPCVSSSGTFPCPQEDSYGNWGCKGMGACSNSQGIAYWSTDLFGFY
Consensus  MTMPHRYMFLAVFTLLALinVASGATEACLPAGQRKSGMNINFYQYSLKDSSTYSNAAYMAYGYASKTKLGSVGGQTDISIDYNIPCVSSSGTFPCPQEDSYGNWGCKGMGACSNSQGIAYWSTDLFGFY

           131      140       150       160       170       180       190       200       210       220       230       240       250      260
           |--------+---------+---------+---------+---------+---------+---------+---------+---------+---------+---------+---------+---------|
Flo1S288C  TTPTNVTLEMTGYFLPPQTGSYTFKFATVDDSAILSVGGATAFNCCAQQQPPITSTNFTIDGIKPWGGSLPPNIEGTVYMYAGYYYPMKVVYSNAVSWGTLPISVTLPDGTTVSDDFEGYVYSFDDDLSQ
Flo1Sigma  TTPTNVTLEMTGYFLPPQTGSYTFSFATVDDSAILSVGGSIAFECCAQEQPPITSTNFTINGIKPWHGSLPDNIAGTVYMYAGFYYPMKIVYSNAVSWGTLPISVTLPDGTTVSDDFEGYVYTFDNNLSQ
Consensus  TTPTNVTLEMTGYFLPPQTGSYTFkFATVDDSAILSVGGaiAF#CCAQ#QPPITSTNFTI#GIKPWgGSLPdNIaGTVYMYAG%YYPMK!VYSNAVSWGTLPISVTLPDGTTVSDDFEGYVYsFD##LSQ

           261      270       280       290       300       310       320       330       340       350       360       370       380      390
           |--------+---------+---------+---------+---------+---------+---------+---------+---------+---------+---------+---------+---------|
Flo1S288C  SNCTVPDPSNYAVSTTTTTTEPWTGTFTSTSTEMTTVTGTNGVPTDETVIVIRTPTTASTIITTTEPWNSTFTSTSTELTTVTGTNGVRTDETIIVIRTPTTATTAITTTEPWNSTFTSTSTELTTVTGT
Flo1Sigma  SNCTIPDPSNYTASTTITTTEPWTGTFTSTSTEMTTITGTNGVPTDETIIVVRTPTTASTIITTTEPWTGTSTSTSTELTTVTGTNGLPTDETIIVIRTPTTASTIITTTEPWT----------------
Consensus  SNCT!PDPSNYaaSTTiTTTEPWTGTFTSTSTEMTT!TGTNGVPTDET!IV!RTPTTASTIITTTEPWngTfTSTSTELTTVTGTNGlrTDETIIVIRTPTTAsTaITTTEPWn................

           391      400       410       420       430       440       450       460       470       480       490       500       510      520
           |--------+---------+---------+---------+---------+---------+---------+---------+---------+---------+---------+---------+---------|
Flo1S288C  NGLPTDETIIVIRTPTTATTAMTTTQPWNDTFTSTSTELTTVTGTNGLPTDETIIVIRTPTTATTAMTTTQPWNDTFTSTSTELTTVTGTNGLPTDETIIVIRTPTTATTAMTTTQPWNDTFTSTSTEIT
Flo1Sigma  ----------------------------------------------------------------------------------------------------------------------------------
Consensus  ..................................................................................................................................

           521      530       540       550       560       570       580       590       600       610       620       630       640      650
           |--------+---------+---------+---------+---------+---------+---------+---------+---------+---------+---------+---------+---------|
Flo1S288C  TVTGTNGLPTDETIIVIRTPTTATTAMTTPQPWNDTFTSTSTEMTTVTGTNGLPTDETIIVIRTPTTATTAITTTEPWNSTFTSTSTEMTTVTGTNGLPTDETIIVIRTPTTATTAITTTQPWNDTFTST
Flo1Sigma  ----------------------------------DTSTSTSTELTTVTGTNGLPTDETIIVIRTPTTASTAITTTEPWNSTFTSTSTEMTTVTGTNGLPTDETIIVIRTPTTASTIITTTEPWTGTSTST
Consensus  ..................................DTfTSTSTE$TTVTGTNGLPTDETIIVIRTPTTAsTAITTTEPWNSTFTSTSTEMTTVTGTNGLPTDETIIVIRTPTTAsTaITTT#PWndTfTST

           651      660       670       680       690       700       710       720       730       740       750       760       770      780
           |--------+---------+---------+---------+---------+---------+---------+---------+---------+---------+---------+---------+---------|
Flo1S288C  STEMTTVTGTNGLPTDETIIVIRTPTTATTAMTTTQPWNDTFTSTSTEITTVTGTTGLPTDETIIVIRTPTTATTAMTTTQPWNDTFTSTSTEMTTVTGTNGVPTDETVIVIRTPTSEGLISTTTEPWTG
Flo1Sigma  STELTTVTGTNGLPTDETIIVVRTPTTAST------------------------------------------------------------------------------------------IITTTEPWTG
Consensus  STE$TTVTGTNGLPTDETIIV!RTPTTAsT..........................................................................................IiTTTEPWTG

           781      790       800       810       820       830       840       850       860       870       880       890       900      910
           |--------+---------+---------+---------+---------+---------+---------+---------+---------+---------+---------+---------+---------|
Flo1S288C  TFTSTSTEMTTVTGTNGQPTDETVIVIRTPTSEGLVTTTTEPWTGTFTSTSTEMTTITGTNGVPTDETVIVIRTPTSEGLISTTTEPWTGTFTSTSTEMTTITGTNGQPTDETVIVIRTPTSEGLISTTT
Flo1Sigma  TFTSTSTEMTTVTGTNGQPTDETVIVIKTPTSEGPISTTTEPWTGTFTSTSTEMTTVTGTNGLPTDETVIIIKTPTTASTIITTTEPWNGTFTSTSTEMTTVTGTNGQPTDETFIVVKTPTTANTIITTT
Consensus  TFTSTSTEMTTVTGTNGQPTDETVIVIrTPTSEGl!sTTTEPWTGTFTSTSTEMTT!TGTNGlPTDETVI!IrTPTsaglIiTTTEPWnGTFTSTSTEMTT!TGTNGQPTDETfIV!rTPTsanlIiTTT

           911      920       930       940       950       960       970       980       990       1000      1010      1020      1030     1040
           |--------+---------+---------+---------+---------+---------+---------+---------+---------+---------+---------+---------+---------|
Flo1S288C  EPWTGTFTSTSTEMTHVTGTNGVPTDETVIVIRTPTSEGLISTTTEPWTGTFTSTSTEVTTITGTNGQPTDETVIVIRTPTSEGLISTTTEPWTGTFTSTSTEMTTVTGTNGQPTDETVIVIRTPTSEGL
Flo1Sigma  EPWTGTFTSTSTEMTTVTGTNGLPTDETVIVVKTPTTANTIVTTTEPWTGTFTSTSTEMTTVTGTNGLPTDETVIVVKTPTTANTIITTTEPWTGTFTSTSTEMTTVTGTNGQPTDETVIVVKTPTT-AI
Consensus  EPWTGTFTSTSTEMThVTGTNGlPTDETVIV!rTPTsanlIsTTTEPWTGTFTSTSTEmTT!TGTNGqPTDETVIV!rTPTsanlIiTTTEPWTGTFTSTSTEMTTVTGTNGQPTDETVIV!rTPTs.ai

           1041     1050      1060      1070      1080      1090      1100      1110      1120      1130      1140      1150      1160     1170
           |--------+---------+---------+---------+---------+---------+---------+---------+---------+---------+---------+---------+---------|
Flo1S288C  VTTTTEPWTGTFTSTSTEMSTVTGTNGLPTDETVIVVKTPTTAISSSLSSSSSGQITSSITSSRPIITPFYPSNGTSVISSSVISSSVTSSLFTSSPVISSSVISSS----TTTSTSIFSESSKSSVIPT
Flo1Sigma  SSSLSSSSSGQITSSITSSRPII---------TPFYPSNGTSVISSSVISSSD---TSSLVTSSLVTSSLVTS---SLVTSSVISSSVTSSLVTSSPVISSSFISSSVISSTTTSTSIFSESSKSSVIPT
Consensus  ssslsepssGqiTSsiTemrp!i.........TpiypkngTsaISSSliSSSd...TSSitsSrl!isplypS...Sl!sSSVISSSVTSSLfTSSPVISSSfISSS....TTTSTSIFSESSKSSVIPT

           1171     1180      1190      1200      1210      1220      1230      1240      1250      1260      1270      1280      1290     1300
           |--------+---------+---------+---------+---------+---------+---------+---------+---------+---------+---------+---------+---------|
Flo1S288C  SSSTSGSSESETSSAGSVSSSSFISS------ESSKSPTYSSSSLPLVTSATTSQETASSLPPATTTKTSEQTTLVTVTSCESHVCTESISPAIVSTATVTVSGVTTEYTTWCPISTTETTKQTKGTTEQ
Flo1Sigma  SSSTSGSSESERSSAGSVSSSSFISSSSFISSESSKSPTYSSSSLPLVTSATTSQETASSLPPSTTTKTSEQTTLVTVTYCESHVCTESISPAIVSTATVTVSGVTTEYTTWCPISTTETTKQTKGTTEQ
Consensus  SSSTSGSSESErSSAGSVSSSSFISS......ESSKSPTYSSSSLPLVTSATTSQETASSLPPaTTTKTSEQTTLVTVTsCESHVCTESISPAIVSTATVTVSGVTTEYTTWCPISTTETTKQTKGTTEQ

           1301     1310      1320      1330      1340      1350      1360      1370      1380      1390      1400      1410      1420     1430
           |--------+---------+---------+---------+---------+---------+---------+---------+---------+---------+---------+---------+---------|
Flo1S288C  TTETTKQTTVVTISSCESDVCSKTASPAIVSTSTATINGVTTEYTTWCPISTTESRQQTTLVTVTSCESGVCSETASPAIVSTATATVNDVVTVYPTWRPQTANEESVSSKMNSATGETTTNTLAAETTT
Flo1Sigma  TTETTKQTTVVTISSCESDVCSKTASPAIVSTSTATINGVTTKYTTWCPISTTESRQQTTLVTVTSCESGVCSETASPAIVSTATATVNDVVTVYPTWRPQTTNEESVSSKMNSATSETTTNTVAAETTT
Consensus  TTETTKQTTVVTISSCESDVCSKTASPAIVSTSTATINGVTTeYTTWCPISTTESRQQTTLVTVTSCESGVCSETASPAIVSTATATVNDVVTVYPTWRPQTaNEESVSSKMNSATgETTTNTlAAETTT

           1431     1440      1450      1460      1470      1480      1490      1500      1510      1520      1530      1540      1550 1556
           |--------+---------+---------+---------+---------+---------+---------+---------+---------+---------+---------+---------+-----|
Flo1S288C  NTVAAETI---------TNTGAAETKTVVTSSLSRSNHAETQTASATDVIGHSSSVVSVSETGNTKSLTSSGLSTMSQQPRSTPASSMVGYSTASLEISTYAGSANSLLAGSGLSVFIASLLLAII
Flo1Sigma  NTGAAETTINTGAAETTTNTGAAKTKTVVTSSLSRSNHAETQTASATDVIGHNNSVVSVSETGNTKSLTSSGLSTMSQQPRSTPASSMVGYSTASLEISTYAGSANSLLAGSGLSVFIASLLLAII
Consensus  NTgAAETi.........TNTGAAeTKTVVTSSLSRSNHAETQTASATDVIGHnnSVVSVSETGNTKSLTSSGLSTMSQQPRSTPASSMVGYSTASLEISTYAGSANSLLAGSGLSVFIASLLLAII
```

Figure S2
